# Special Issue: Type III Secretion Systems in Human/Animal Pathogenic Bacteria

**DOI:** 10.3390/microorganisms10071461

**Published:** 2022-07-20

**Authors:** Joaquín Bernal-Bayard, Francisco Ramos-Morales

**Affiliations:** Departamento de Genética, Facultad de Biología, Universidad de Sevilla, 41012 Sevilla, Spain; jbbayard@us.es

Type III secretion systems (T3SSs) are molecular devices that are essential for the communication of many Gram-negative bacteria with their eukaryotic hosts [1]. They were initially described, some 30 years ago, in bacteria from the genera *Yersinia* and *Salmonella* [2,3], but are also present in many other Gram-negative bacteria including *Aeromonas*, *Bordetella*, *Burkholderia*, *Chlamydia*, *Citrobacter*, *Edwardsiella*, *Erwinia*, *Escherichia*, *Photorhabdus*, *Pseudomonas*, *Ralstonia*, *Rhizobium*, *Shigella*, *Vibrio* and *Xanthomonas* [4,5].

The structure of these multiprotein molecular machines, also known as injectisomes, has been studied in detail. It contains more than 20 proteins and includes a cytoplasmic sorting platform, an inner membrane export apparatus, a basal body, a needle complex, and a translocation pore in the host cell membrane [6,7,8,9,10].

At least eight subfamilies of injectisomes can be distinguished: Ysc, SPI-1, SPI-2, and the *Chlamydia* subfamily, which are present predominantly in animal pathogens; Hrp1, Hrp2, and the *Rhizobium* subfamily, present in plant pathogens; and the *Myxococcus* subfamily [11].

Using these systems bacterial pathogens and symbionts are able to inject proteins, known as effectors, into the cytosol of animal, plant, fungi, or protist cells [12]. These effectors may interact with specific components of the host to alter their behavior and interfere with signal transduction pathways to establish an appropriate survival niche. Although the physical interaction with a host target may be the main mechanism of action for some effectors, many effectors possess enzymatic activities that include guanine nucleotide activating, cysteine protease, acyltransferase, deubiquitinase, kinase, AND-ribosyl transferase, phosphothreonine lyase, zinc metalloprotease, glycosyltransferase, and ubiquitin ligase [13].

This Special Issue focuses on the study of type III secretion systems in animal pathogenic bacteria. Despite the variety of lifestyles and diseases caused by these pathogens, there are also common themes arising from the fact that they share the use of T3SSs as key virulence mechanisms, and that effectors from different bacteria may have similar designs, biochemical activities, and/or functions. Therefore, researchers working in a particular T3SS can benefit from the studies carried out on other bacteria.

The Special Issue “Type III secretion systems in human/animal pathogenic bacteria” gathers nine papers covering studies in bacteria from the genera *Aeromonas*, *Citrobacter*, *Edwardsiella*, *Escherichia*, *Salmonella*, and *Shigella*.

Working on *Edwardsiellla ictaluri*, which causes enteric septicemia of catfish, Dubytska and Thune [14] described in detail the development of the *Edwardsiella* containing vacuole (ECV) in infected macrophages. Immunofluorescence of vacuolar membrane markers shows that, although the recruitment of the early endosomal markers Rab5 and EEA1 and the late endosomal marker Rab7 is similar in ECVs from a wild-type strain and a T3SS-mutant strain, the differential colocalization of Lamp1 indicates that the T3SS is essential to prevent phagosomal/lysosomal fusion. This secretion system is also important to suppress autophagy and apoptosis in macrophages.

Barkalita et al. [15] developed a reporter system based on PhoA activity to monitor secretion through the injectisome of enteropathogenic *Escherichia coli*. Pendergrass et al. [16] describe another reporter system based on the secretion of the enzyme carboxypeptidase G2 that, after delivery cleaves the substrate Glu-CyFur, releases the fluorescent molecule CyFur, and results in a visible color change from yellow to purple. These authors show that this system is useful to detect T3SS inhibitors.

The fish pathogen *Aeromonas salmonicida* possesses a T3SS that is essential for its virulence and is encoded in the plasmid pAsa5. The region encoding this system may be lost during growth at 25 °C due to recombination between insertion sequences present on the plasmid, but there is great variability between strains for this phenomenon. A paper in this Special Issue by Marcoux et al. explored this variability in 50 strains of *A. salmonicida* subsp. *salmonicida* and performed a comparative genomic analysis to identify genes related to the molecular mechanism involved [17].

Single cell analysis is also represented in this Issue with a study carried out by Sánchez-Romero and Casadesús in *Salmonella enterica* that allowed the identification of four subpopulations depending on the expression of *Salmonella* pathogenicity island I and/or the flagellar regulon [18].

Fernández et al. [19] report the construction of plasmids that are useful to generate chromosomal CyaA’ translational fusions by homologous recombination using the Red recombination system. These fusions can be used to measure the level of expression of a gene and the secretion of a fusion to the culture supernatant by Western blot using anti-CyaA’ antibodies. Also, since CyaA’ from *Bordetella pertussis* is a calmodulin-dependent adenylate cyclase that is only active in eukaryotic cells, these fusions can be used to detect the translocation of an effector into host cells.

These research articles are complemented by three review papers. A review by Araujo-Garrido et al. [20] focuses on a family of effectors with arginine N-glycosyltransferase activity. This family includes NleB from *Citrobacter rodentium*, NleB1 and NleB2 from enteropathogenic and enterohemorrhagic *E. coli*, and SseK1, SseK2, and SseK3 from *S. enterica*.

Finally, the T3SS of *Shigella* is reviewed by two leading groups in the field. In addition, to describe the structure, assembly, and function of the system, both groups emphasize different aspects related to it. Muthuramalingam et al. [21] highlight the possibility of using the T3SS as a therapeutic target for small molecules or vaccines. Bajunaid et al. [22] focus on the role of the system in the escape of *Shigella* from the vacuole.

Altogether, the papers of this Special Issue give to interested readers an idea of the diversity and common themes of these fascinating nanomachines in animal pathogens.
